# The effect of L-PRF membranes on bone healing in rabbit tibiae bone defects: micro-CT and biomarker results

**DOI:** 10.1038/srep46452

**Published:** 2017-04-12

**Authors:** Fernanda Faot, Sanne Deprez, Katleen Vandamme, Germana V. Camargos, Nelson Pinto, Jasper Wouters, Joost van den Oord, Marc Quirynen, Joke Duyck

**Affiliations:** 1 Department of Restorative Dentistry, Prosthodontics Division, Graduate Program in Dentistry, School of Dentistry, Federal University of Pelotas, Rio Grande do Sul, Brazil; 2 KU Leuven & University Hospitals Leuven, Department of Oral Health Sciences, BIOMAT KU Leuven & Prosthetics section, Leuven, Belgium; 3 Department of Dental Materials and Prosthodontics, Federal University of Alfenas, Minas Gerais, Brazil; 4 Department of Surgical and Prosthetic Implantology, Faculty of Odontology, University of the Andes (UANDES), Santiago, Chile; 5 Department of Imaging & Pathology, Translational Cell & Tissue Research, KU Leuven, Belgium; 6 KU Leuven & University Hospitals Leuven, Department of Oral Health Sciences, BIOMAT KU Leuven & Periodontology, Leuven, Belgium

## Abstract

More insight into the biological fundamentals of leukocyte platelet-rich fibrin (L-PRF) guided healing is necessary to recommend its application, in particular in deficient bone sites that need to support implants. This study investigated the short-term bone healing effect of L-PRF treatment in cylindrical non-critical sized bone defects with 3 mm diameter and 6 mm depth in tibiae of 18 adult male New Zealand White rabbits. After a randomization process, 96 bone defects were prepared and half of them were filled with a L-PRF membrane, while untreated defects in the opposite tibia served as control group. The rabbits were euthanized after 7, 14 or 28 days of healing. The bone healing of the cortical and medullary areas was investigated by micro-CT, while the expression of molecular markers (RUNX2, VEGFA, COL1A2 and BMP2) was assessed by qRT-PCR. Treatment with L-PRF did not affect the micro-structural bone characteristics of the repaired bone tissue, except for a decrease in the trabecular connectivity at the cortical level after 14 days of healing. At this time, RUNX2 and VEGFA mRNA levels were significantly lower in the treated defects. L-PRF membranes thus had a temporary negative influence on the bone microarchitecture (Tb.Pf) and on the RUNX2 and VEGFA expression during early bone healing. Overall, L-PRF treatment did not enhance bone regeneration in these non-critical size defects after 28 days.

In many cases involving extended periods of tooth loss, alveolar bone grafting is required before or during oral implant placement. Sinus lift techniques and socket preservation procedures require biomaterials capable of inducing fast and effective wound healing and bone regeneration^1^. Since autologous bone grafting is associated with considerable morbidity and the single use of biomaterials still requires long healing processes^2^, platelets concentrates are intensively investigated as an alternative method for improved bone regeneration^3^.

Platelet rich fibrin (L-PRF) was first described by Choukroun *et al*. (2001), and has been referred to as a second generation platelet concentrate^4^. Fibrin is a natural guide for angiogenesis, traps the circulating stem cells^5^ and provides wound protection by epithelial coverage^6^,^7^. The fibrin matrix of L-PRF is obtained as a result of slow polymerization. This matrix can hold many growth factors and cytokines, and can release them in the wound site for prolonged times^8^.

The growth factors present in L-PRF have been shown to accelerate bone repair and to promote fibroblast proliferation^9^. In addition, these factors increase tissue vascularity, the rate of collagen formation, and mitosis of mesenchymal stem cells, endothelial cells and osteoblasts^9^. Several authors have also demonstrated that a fibrin matrix provides an optimal support for mesenchymal stem cells^6^,^10^,^11^, which contribute to regeneration of osseous defects and of many other tissues. As a consequence of all these powerful effects on tissue regeneration, a growing number of human clinical studies have confirmed the beneficial effect of the use of growth factors via L-PRF application in reconstructive oral and maxillofacial surgery^12^, including periodontal surgery^13^,^14^,^15^,^16^ implants^17^ and sinus grafting^18^,^19^.

So far, studies that investigated the biologic events in the early stages of L-PRF -mediated bone healing are limited and restrict to the cortical bone^2^,^20^. Most studies involved the use of L-PRF for soft tissue repair^21^, while the use of its fibrin-based material solely as a filling material for large bone defects remains controversial. One study^22^ reported positive effects of L-PRF when used in conjunction with titanium barriers. L-PRF use in the latter case increased the quality of the newly formed bone and enhanced the rate of bone formation, attributed to the concentration of growth factors. Kim *et al*.^1^ also reported increased bone mineral density and bone volume in calvaria bone defects of rabbits treated with PRF already after 6 weeks. These results were similar to two other platelets concentrates tested: PRP and Concentrated Growth Factor. Cho *et al*.^23^ reported a positive bone healing effect of L-PRF on bone integration (higher bone density) of oral implants installed at 4 weeks after preparing bone defects in rabbit tibias, using removal torque measurements. The animal study conducted by Knapen *et al*.^2^ on the other hand, showed that L-PRF did not provide any additional effect on the kinetics, quality and quantity of bone during guided bone regeneration over a period of 12 weeks.

The present study aimed to evaluate the effect of L-PRF on the early stages of bone healing using an *in vivo* rabbit model. The study was set up to test the hypothesis that the treatment of non-critical sized bone defects by L-PRF application enhances the bone healing dynamics. Bone repair was investigated with or without L-PRF membranes applied in non-critical sized long bone defects in rabbits using microCT, after healing periods of 7, 14 and 28 days. Expression of molecular markers for bone healing, BMP2, RUNX2, VEGFA and COL1A2, was assessed by qRT-PCR.

## Methods

### Experimental design

The study was designed as a randomized test-control study. Eighteen male New Zealand White Rabbits weighing between 3 and 4 kg were selected for this study. The number of animals required was estimated on the basis of previous studies^1^,^2^,^24^. A minimum of 12 samples (bone defects) was needed to detect a difference between 2 groups (power of 80% and an error probability of 5%). Our study considered 16 as the sample size of each group (with or without L-PRF membranes) resulting in 6 samples to be evaluated according to the periods of time: 7, 14 and 28 days. We also planned our experimental design taking in account the Effect Size evaluation, once we adopted the animal as its own control. Effect sizes found in laboratory studies are normally high, especially in paired studies, since the intra-animal variation is likely less than the inter-animal variation. To achieve this, 18 rabbits were divided into 3 groups and each rabbit should receive at least 4 bone defects (2 L-PRF, 2 control) for CT and molecular analysis, respectively. The observed effect sizes ranged between 0 and 1.9, and our study detects large effect sizes of 1.8 with 80% confidence, at the conventional significance level of 5%.

The experimental protocol was approved by the KU Leuven University Committee on Use and Care of Animals. All methods in this study were performed in accordance with the relevant guidelines and regulations as provided by this committee. The manuscript was prepared according to ARRIVE guidelines^25^. The rabbits were kept in separate cages and their diet consisted of standard rabbit food pellets and water, available *ad libitum.* They were housed in a room maintained at 24 °C with a 12-h light/dark cycle. The animal’s weight was monitored weekly. A total of 36 bilateral tibias were randomized taking in account the test and control sides, as well as, the bone defect position for the microCT and qRTPCR analysis.

The bone defects were left to heal for 7, 14 or 28 days (n = 6 for each healing time point). In total, 48 samples were created for each condition. The 12 rabbits where the defects were allowed to heal for 7 or 14 days received 6 bone defects: 3 test defects in one tibia and 3 control defects in the other tibia. The 6 remaining rabbits of the 28 days healing period received only 4 defects (2 per tibia). Per rabbit a test and control sample were intended for micro-CT and histological analyses. Micro-CT analysis was performed in 6 samples of each group to evaluate the bone micro-architecture of control and test defects at the mentioned healing times. qRT-PCR analysis was performed only for the 7 and 14 days healing to investigate the expression of genes that are typically expressed during the early bone healing.

### Bone defect preparation

**Figure 1:**
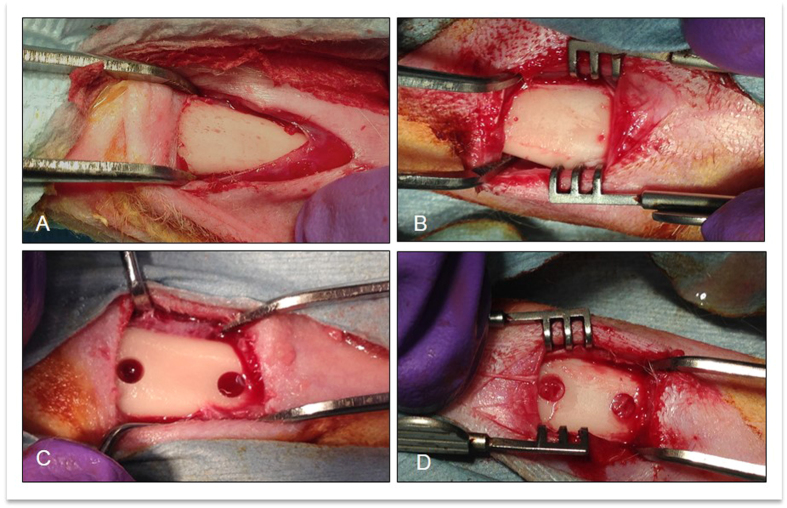
Surgical steps during bone defect creation and L-PRF insertion. (**A**) Tibia exposition; (**B**) Marking the planned bone defect areas; (**C**) Bone defect creation at 10 mm inter-distance (measured between the centers of the bone defects); (**D**) Bone defects filled with L-PRF membrane.

After uncovering the medial part of the proximal diaphysis of both tibiae, 2 or 3 cylindrical bone defects were drilled in both tibiae. In one tibia, the defects were filled with L-PRF (test defects) whereas the defects in the contralateral tibia remained unfilled (control defects). The bone defects were made using a 3-mm trephine bur (Medicon CMS, Tuttlingen, Germany), thereby creating 3-mm wide cylindrical defects. The drill perforated the medial cortex and was inserted until it touched the inner side of the lateral cortex resulting in a defect length of approximately 6 mm. Each tibia received several bone defects (2 or 3), with 10 mm inter-distance (measured between the centers of the bone defects) (Fig. 1). The skin was sutured (Vicryl 3/0, Ethicon, Johnson and Johnson, USA) and the animals were given 0.015 mg/kg of buprenorphine (Vetergesic, Ceva, Belgium) as pain relief. The bone defects were left to heal for 7, 14 or 28 days.

### L-PRF membrane preparation

**Figure 2:**
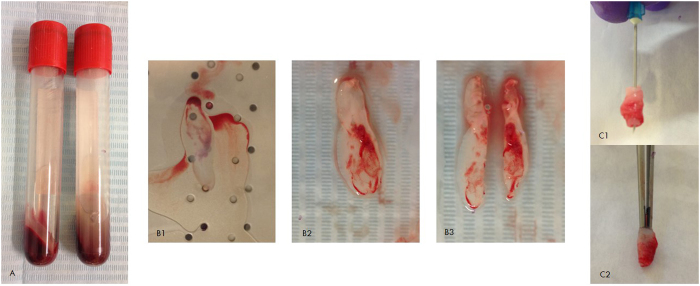
(**A**) L-PRF generated after centrifugation, the volume of 10 mL of blood collection was distributed in 2 tubes; (**B**) 1. Squeezing of L-PRF clot; 2. L-PRF membrane; 3. Splitting the L-PRF membrane. (**C**) The L-PRF membrane before insertion into a cavity.

For each L-PRF membrane, 10 ml of blood was collected from the central vein of the rabbit ear with a syringe, transferred 2 aliquots of 5 ml to plastic tubes and immediately centrifuged in a small laboratory centrifuge (Model EBA 20, Andreas Hettich GmbH&Co. KG). The standard centrifugation parameters were 2700 RPM for a period of 12 minutes^4^. This protocol was able to successfully produce L-PRF (Fig. 2), as observed in the pilot study. The L-PRF clot was subsequently compressed with a glass plate for 4 minutes, and 2 L-PRF membranes were produced with similar dimensions^26^. Prior to filling the defect, the L-PRF membrane was wrapped and the leucocyte-rich blood interface was exposed in order to be positioned inside of the cavity in contact with the bone defect walls.

### Post-operative monitoring

After surgery, all the animals were housed at room temperature 18 ± 3 °C and relative humidity (55 ± 15%). The feeding condition, weight, body temperature, breathing, appearance of surgical site, the possibility of incision infection, the movement function (animal’s ability to ambulate and perform nomal body movements), pain and distress of rabbits were monitored daily during the first week, each 3 days during the second week and weekly after the third week until the study completion.

### Specimen preparation for micro-X-ray computed tomography analysis

After euthanizing the animals with an i.v injection of 2 mL of T61 (embutramide-mebenzoniumjodide-tetracaine HCl solution), the tibia skin was excised and the defect sites were removed along with surrounding bone tissue, and immediately fixated in 10% CaCO_3_–buffered formalin solution (pH 7.4) at 4 °C for 48 h. The samples were then preserved in 70% ethanol at 4 °C in attendance of the micro-X-ray computed tomography (μCT) scanning. One control and 1 test sample per rabbit was used for micro-CT analysis.

**Figure 3:**
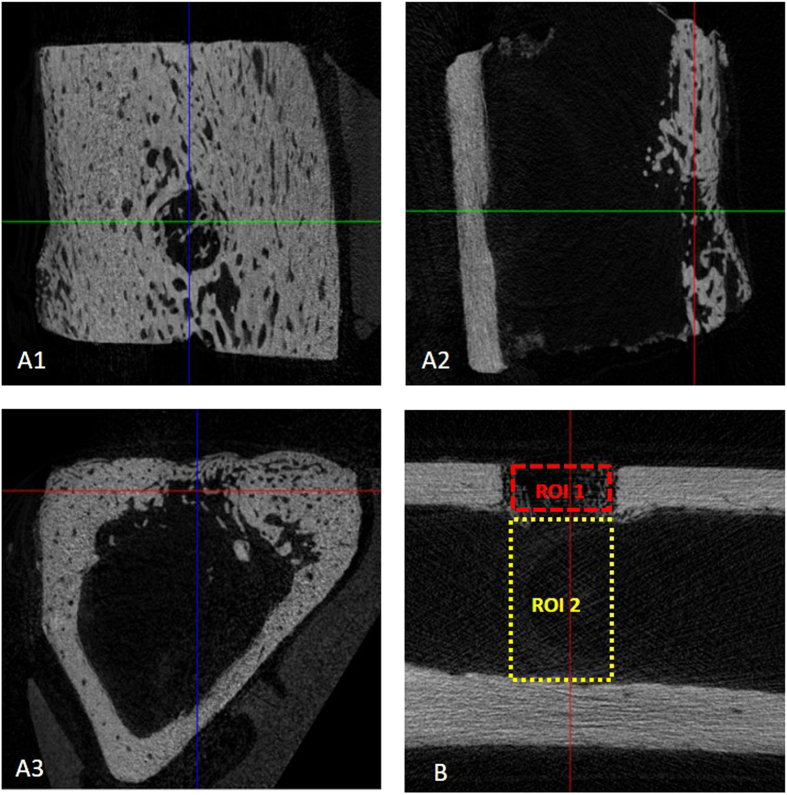
(**A**) Bone defect localization in the 3 planes orientation to explore bone defect (Data viewer software, Bruker, Kontich, Belgium); (**B**) Regions of interest analyzed: ROI 1 (Cortical area, 3 × 1 mm) and ROI 2 (Medullary area 3 × 5.2 mm).

To assess the bone micro-architectural changes in response to L-PRF membrane application (test) compared to no L-PRF application (control), 1 test and 1 control sample were examined *ex vivo* using a Skyscan 1172 μCT system (Bruker, Kontich, Belgium). During scanning, the tibia was protected by a parafilm pellicle and immobilized onto the sample supporter by means of a soft modeling clay. The bone samples were scanned along the longitudinal planes in the medial and lateral regions to obtain the μCT images. The following scanning parameters were used: 14.1 μm pixel size, 50 kV X-ray voltage, 200 μA electric current and 0.5 mm Al filter. The scanning resulted in reconstructed 3D data sets with a voxel size of 14.1 μm, which were subsequently explored in the Dataviewer software and quantified using a CTAn automated image analysis system (Bruker, Kontich, Belgium) (Fig. 3A). Volumes of interest were defined at trabecular and at cortical level as detailed below. The morphometric parameters were calculated according to the methodology described by Bouxein and co-workers (2010)^27^.

To determine the volume of interest in the axial direction, the dimensions and shape of the bone defect were used as a reference to localize the regenerated area. For the morphometric analysis, a subregion of the original dataset was selected for a representative measurement. The selected region of interest (ROI) included the whole defect area containing the newly formed bone (Fig. 3B). By auto-interpolation between layers, the ROI became the volume-of-interest (VOI), which is the essential basis for the 3D quantitative analysis. A VOI with a rectangular shape was determined in the transaxial plane with a fixed width of 3 mm and 2 different height dimensions: the VOI for the cortical area was 1 mm, whereas the height of the VOI rectangle for the medullary area was between 5 and 6 mm considering the distance between the outside surface of the medial cortical (i.e. site of the periosteum) and the inside surface of the lateral cortical bone (i.e. site of the endosteum). Images from the axial projection of the tibiae were taken, which resulted in 212 layers covering the whole defect. Within the VOI, the volume of the newly formed bone was calculated as a percentage. Based on the calculated histogram, the bone was defined to be in the 64–225 gray value range.

The following bone micro-structural parameters of newly formed bone were evaluated at both the cortical and trabecular (medullar) level: Bone volume (BV, mm^3^); Bone volume/Tissue volume (BV/TV, %); Bone Surface (BS, mm^2^); Intersection surface (i.S, mm^2^); Bone surface/Volume ratio (BS/BV, mm^−1^); Trabecular pattern factor (Tb.Pf, mm^−1^); Structure model index (SMI); Degree of ansiotropy (DA); Fractal Dimension (FD). Furthermore, Tissue volume (TV, mm^3^); Tissue Surface (TS, mm^2^); Bone surface density (BS/TV, mm^−1^); Trabecular thickness (Tb.Th, mm); Trabecular separation (Tb.Sp, mm) and Trabecular number (Tb.N, mm^−1^) were also evaluated at the level of trabecular ROI.

### RNA extraction, complementary DNA (cDNA) synthesis and quantitative real time PCR (qRT-PCR)

After euthanizing the animals, bone defect samples for qRT-PCR were immediately harvested and submerged in a nontoxic tissue storage reagent (RNAlater solution, Thermo Scientific, USA) and were frozen at −20 °C for later processing. RNA was extracted from 24 samples of the 7 day and 14 day groups (12 samples per group; 1 test and 1 control sample from each rabbit) using the TRIzol reagent (Invitrogen, Thermo Fisher Scientific, Waltham, USA) according to manufacturer’s instructions. Briefly, bone samples were grinded in a mortar with TRIzol reagent and incubated at room temperature for 5 minutes to lyse the cells. Chloroform was added to separate the samples into a phenolchloroform phase, an interphase, and an aqueous phase. The latter contains the RNA and was used for the RNA isolation step. RNA was precipitated by addition of isopropanol. The resulting precipitate was washed with ethanol and resuspended in RNase-free water. The concentration of purified RNA was measured on the NanoDrop ND-1000 Spectrophotometer (NanoDrop, Thermo Fisher Scientific)

cDNA was synthesized from 500 ng of RNA using Superscript II Reverse Transcriptase (RT) (SS II kit, Invitrogen). Briefly, 0.8X random primers (Invitrogen) and 0.8 mM deoxyribonucleotide triphosphates (dNTP mix, Invitrogen) were added and the samples were heated to 65 °C for 5 minutes and afterwards cooled on ice. During this cooling period, a mixture of 1x First Strand Buffer (Invitrogen), 0.01 M 1,4-dithio-DL-threitol (DTT), 5% RNase OUT (Invitrogen) and 10 units of Superscript II RT was added. Lastly, the samples were heated to 25 °C for 10 minutes, 42 °C for 50 minutes and 70 °C for 2 minutes, to perform the subsequent steps of reverse transcription. Afterwards, the samples were cooled on ice.

Gene expression levels were analysed using the qRT-PCR method. Rabbit-specific primer-probe sets were commercially acquired (TaqMan Gene Expression Assays, Applied Biosystems, Thermo Fisher Scientific) for glyceraldehyde 3-phosphate dehydrogenase (GAPDH, Oc03823402_g1), β-actin (ACTB, Oc03824857_g1), Runt-related transcription factor 2 (RUNX2, Oc02386741_m1), bone morphogenetic protein 2 (BMP2, Oc03824113_s1), vascular endothelial growth factor A (VEGFA, Oc03395999_m1) and collagen type 1 alpha 2 (COL1A2, Oc03396455_m1). TaqMan Gene Expression Master Mix 1x (Applied Biosystems) and cDNA (0.3 μg) were added to the primer-probe mix. The procedure was completed using the 7900 HT Fast Real-Time PCR System (Applied Biosystems). The samples were heated to 50 °C for 2 minutes, then brought to 95 °C for 10 minutes, followed by 50 cycles of 15 seconds at 95 °C and 1 minute at 60 °C. During the last step of each cycle, the fluorescent signal was measured. Expression levels were analysed using the ΔΔCt method, after normalization against the pooled expression levels of housekeeping genes GAPDH and ACTB.

### Statistical Analysis

Statistical analyses were performed using the SAS v. 9.0 software (SAS Institute, Inc, Cary, NC) and R v3.2.3 (R Core Team, Vienna, Austria) with a significance level fixed at 5%. The assumptions of equality of variances and normal distribution of errors were evaluated for each variable, and the data were logarithmically transformed when these assumptions were violated. The values of the microarchitectural parameters and mRNA expression levels were compared between test and control groups and within the groups for each healing period (7, 14 or 28 days) using 2-way ANOVA and 1-way ANOVA respectively, in order to assess the effect of the treatment and of the healing time, and their interactions with the cortical and medullar bone micro-architectural response to the bone defect surgery. Post-hoc comparisons were done using the Tukey Honestly Significant Difference (HSD) test.

## Results

**Figure 4:**
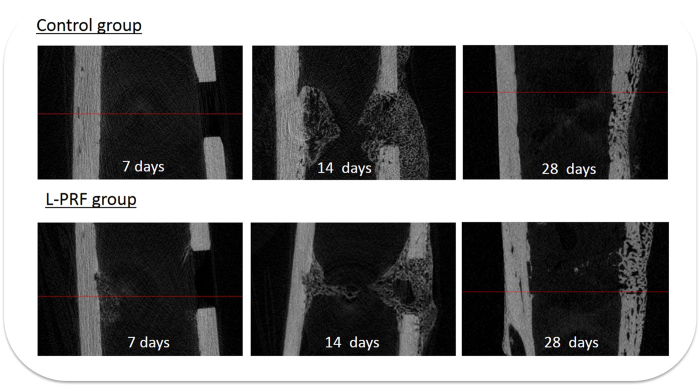
Bone repair morphological characteristics in the cortical and medullary areas according to the healing times.

There were no intra-operative clinical issues; post-operatively all rabbits regained independent feeding ability with normal gait, following recovery from anesthesia. All animals except 1 recovered from surgery without complications and showed no signs of discomfort (apparent signs of infection such as a red, hot incision or exudate) during the healing process. One rabbit of the 7-days group fractured a tibia on the 3rd day post-surgery. The fracture did not involve a bone defect under investigation. The tibia was immobilized, the rabbit received intramuscular injection of buprenorphine (Vetergesic 0.3 mg/ml, dose: 0.05 ml/kg) as analgesic for 4 days, displayed no additional problems, and was sacrificed at day 7. Bone repair morphological characteristics in the cortical and medullary areas can be observed in Fig. 4.

The 2-way ANOVA results of the bone micro-architecture for the different healing periods and for the different experimental conditions (L-PRF application or not) are shown in Table 1 for the cortical and the medullary region.

The healing time effect (*i.e.* 7, 14, and 28 days) was statistically significant for all structural parameters evaluated in the cortical and medullary area (ANOVA *p* < 0.05; Supplementary material), except for the trabecular volume (TV) and trabecular surface (TS) parameters. The results of the control *versus* L-PRF membrane group on the other hand indicate that the treatment did not affect the bone micro-structural parameters, neither at cortical nor at medullar level. The interaction between the independent variables healing time and treatment modality at the cortical bone repair site was positive for the parameter Tb.Pf. For the other cortical level parameters, no interaction was found. (ANOVA *p* < 0.05).

As expected, the duration of healing was the main factor that influenced the bone repair at the cortical level (Table 2). The variables BV, BV/TV, Tb.Pf, SMI and FD for the repaired cancellous bone showed a statistically significant increase (*p* < 0.05) after 28 days of healing compared to the other evaluated healing time points. Statistical differences were found between the control group and L-PRF treatment after 14 days of healing only for the Tb.Pf parameter (p = 0.04) with higher values for the L-PRF treatment. Between 14 and 28 days, the DA and FD values decreased significantly (*p* < 0.05). Finally, significant lowest BV, BS/TV, BS and i.S values (*p* < 0.05) were observed after 7 days healing.

Also at the medullar level, the time of healing was the main factor influencing the bone repair (Table 2). For all medullar level parameters, no interaction was found between the time of healing and the treatment type. Unlike in the cortical area, most of the parameters reached significant highest values after 14 days of healing: BV (p < 0.0001); BV/TV (p < 0.0001); BS (p < 0.0001); BS/BV (p < 0.0001); BS/TV (p < 0.0001); Tb.N (p < 0.0001) and i.S (p = 0.0075), while Tb.Th (p < 0.0001), Tb.Sp (p < 0.0001) and DA (p = 0.0001) the significant lowest values for the 14 days healing time point. After 7 days healing time, the Tb.Pf parameter showed the significantly lowest value (p < 0.0001), while the SMI parameter was significantly highest (p < 0.0001). After a 28 days healing period, significantly highest values were only found for the FD parameter (p = 0.0013).

**Figure 5:**
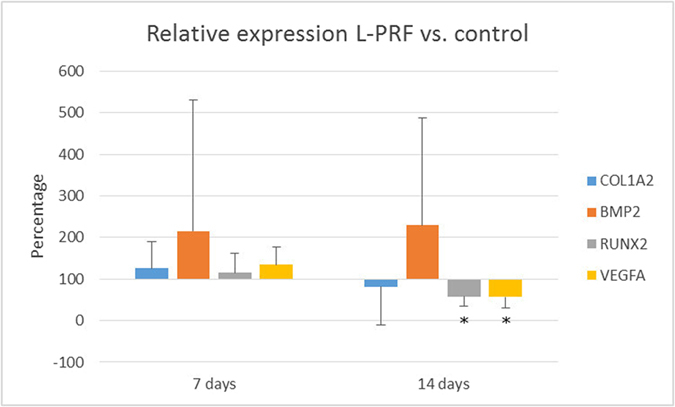
mRNA expression levels of COL1A2, BMP2, RUNX2 and VEGFA. Data are represented as relative expression in L-PRF treated defect compared to control defect with values of the control defect set as 100% expression. (*p < 0.05, n = 6). qPCR analysis was not performed for samples of d28.

The qRT-PCR results are shown in Fig. 5. After 7 days of healing time, no significant differences in expression levels were observed between the L-PRF-treated defect and the control defect. After a healing period of 14 days, the expression levels of RUNX2 and VEGFA were significantly decreased in the treated site compared to the control site.

## Discussion

Taken the results together, the hypothesis that the application of L-PRF in non-critical sized bone defects enhances the bone healing dynamics was rejected. Although the qRT-PCR results show a difference on the mRNA level after 14 days, this difference did not result in a change of the outcome.

L-PRF is in essence a fibrin matrix in which platelets, cytokines and cells are trapped and may be delivered within a certain time frame^28^. Clinically, the use of L-PRF includes advantages as ease of preparation and lack of biochemical handling of blood that makes this preparation strictly autologous. Furthermore, it provides adhesiveness and tensile strength for clot stabilization. Thus, its use as an autologous scaffold for periosteal cells and for osteoblasts has shown to be able to promote higher metabolic activity and cell proliferation^6^,^29^. These positive effects during wound repair are not related only to haemostasis, but also to the fact that it can provide a matrix for migration of tissue-forming cells like fibroblasts and endothelial cells, which are involved in tissue (re)modelling and angiogenesis.

Up to now, the potential for bone formation was only evaluated in calvaria^2^ bone defects, i.e. a cortical bone shell, or in tibia defects in combination with other materials^2^,^24^,^30^,^31^,^32^. Time points for monitoring the healing process in the presence of L-PRF included periods longer than 4 weeks 1,2,33. However, the clinical beneficial effect of L-PRF on bone regeneration during the early stages of bone regeneration is largely unexplored. In the context of the limited information related to the role of L-PRF during early healing, our study evaluated the effect of L-PRF on the early stages of bone regeneration in non-critical sized bone defects, expecting enhanced healing dynamics compared to the normal wound-healing process. With this purpose, the osteoinductive properties of the L-PRF membranes, in contact with exposed cortical and medullary tissues of the tibiae of rabbits were investigated by microCT and qRT-PCR, for healing periods of 7, 14 and 28 days.

Our results indicate that the treatment of non-critical sized bone defects by L-PRF application did not significantly enhance the healing dynamics, at least in terms of bone morphological parameters. The comparison between the control and the L-PRF membrane sites revealed that the treatment did not affect the micro-structural parameters neither in cortical nor in the medullary region, with the exception of a significantly higher bone trabecular parameter (Tb.Pf) in the 14-days L-PRF healing group compared with the control group. The higher Tb.pf values in the L-PRF group indicate a less favourable trabecular geometry developed in the first 2 weeks. The latter suggests an altering effect on bone repair, since a well-developed trabecular structure provides a rigid framework, and forms a good basis for further calcification. However, despite the fact that the trabecular structure in the L-PRF group improved at an accelerated rate between the 2nd and the 4th week, this morphological parameter was not highly significant (p = 0.044) in the comparisons between control and test groups, and identical Tb.pf values were achieved in the end of the study in the control group. Based on the results of this study, we cannot determine whether this trend continues past 28 days.

The bone volumes in the experimental and control groups were similar after 14 and 28 days, although the study by Kim *et al*. found an increased bone volume with L-PRF in rabbit calvaria after 6 weeks^1^. Although improved bone volumes on short time frames were not found in the present study, it could be that the accelerated improvement of the trabecular connectivity in the L-PRF sites after 2 to 4 weeks of healing, continues beyond 4 weeks healing time, thereby leading to improved bone regeneration with L-PRF after longer healing periods. One explanation for the lack of indications for L-PRF-mediated accelerated healing in this study is related to the type of the bone defect adopted in the present study. The performed defects were non-critical sized defects, implying that healing was expected to occur in either case. In addition, the morphology of the tibiae does not favour retention of the L-PRF clot during healing. Since the medullary area is a rather “hollow tube” that is highly vascularised, and filled with soft tissues, the clot is mainly retained by the cortical bone walls. The long-term effect of the L-PRF membrane is difficult to estimate, although one study noted a slow release of growth factors for at least 7 days^8^. In our study, we could also observe that the membrane was not fully resorbed after 14 days (Fig. 4), placing an upper limit on the residence time of the L-PRF clot.

According to Knapen *et al*.^2^, connective cells could be observed in the region of the osteotomy 1 week post-L-PRF application but did not extend into the L-PRF clot. After 5 and 12 weeks no benefits were described in terms of the rate of bone mineralization. So far, positive results for L-PRF are exclusively described in association with titanium barriers^22^, which likely improves the residence time of the L-PRF clots, or when a “critical sized defect” model is used^1^. In our study, the treated group at the 14 days healing time point showed a reduced trabecular connectivity.

Additionally, our study showed no positive effect of L-PRF on mRNA expression of some important factors in bone healing. Currently, no research has been conducted to investigate the effects of L-PRF on mRNA expression during the inflammatory process and bone regeneration in animals. In this study, we chose to investigate RUNX2, VEGFA, BMP2 and COL1A2 because they are the best-known factors involved in bone healing and can influence multiple pathways that are involved in the repair process. In addition, RT-PCR analysis in this small panel of molecular markers was performed in order to identify early subtle changes in gene expression and to investigate their physiological importance when L-PRF membranes are applied. Unfortunately, measuring the protein levels of the investigated growth factors was not possible due to the limited availability of sample tissue.

Our biomarker results showed no positive effect of L-PRF on mRNA expression, and contrary to what we expected, L-PRF application temporally resulted in significantly lower expression of RUNX2 and VEGFA after 14 days. The actual concentration values are low, and it is difficult to assess whether this would result in a physiological difference, although this incidence coincides with inferior trabecular connectivity. The lower RUNX2 and VEGFA values could be explained in 2 ways., Firstly, L-PRF membranes might alter the distinct pattern of growth factor kinetics, as shown in rabbit bone healing by Zhang *et al*.^34^ and Li *et al*.^35^. A clinical study by Eren *et al*.^36^ also found decreased values of early bone repair markers upon L-PRF application. These researchers hypothesized that the healing process in the L-PRF treated group was already in an advanced phase, requiring less biomarkers. A last explanation is that growth factors which are released from L-PRF could interfere with the expression of growth factors in the host tissue by means of inhibition. This might either be because the growth factors released from L-PRF are already at the needed level, so no additional production is necessary by the host tissue. Previous studies investigating application of recombinant growth factors^37^,^38^ have shown that the dose or release kinetics themselves might actually inhibit bone formation. Unfortunately, further research, including measuring the protein levels of the investigated growth factors, was not possible due to the limited availability of sample tissue.

Along with the short term effects of L-PRF treatment, our study provided also some insights into the wound healing in early stages of bone formation, in terms of volumetric changes during bone defect repair and bone neoformation in the cortical and in the medullar bone respectively. We argue that our findings are relevant for future L-PRF studies with animal models. Approximately 60% of the bone defect was healed at the cortical bone level, irrespective of the treatment. Indeed, a bone bridge was not formed by the end of the experiment. Although this seems to favour performing long-term studies, our observed difference in trabecular structure would not have been observed in studies which solely focus on longer healing times. Similarly, the degree of anisotropy and the fractal dimension, 2 parameters closely related to the bone structure, had statistically significant decreased values (p < 0.05) after 14 days compared to the 7 days group, but increased again in the period leading up to 28 days. This highlights the importance of investigating the bone healing process at early time points. These findings show that the early healing period is of critical importance during the bone healing. Also in terms of rabbit behavior, rabbits start to be more mobile 7 days post-surgery, increasing the probability of membrane dislodgement. The aforementioned effects indicate that future studies should consider both short-term and long-term effects, and consider placement of barriers that promote the retention of the L-PRF membranes.

In the medullar region, the duration of healing was also the main factor that influenced bone regeneration. In contrast with the cortical area, most of the parameters reached significantly higher values after 14 days healing: BV; BV/TV; BS; BS/BV; BS/TV; Tb.N i.S. This indicates that bone healing is processed differently in the medullar region, in which greater BV/TV up to 16% was observed after 14 days of healing reducing to 5–10% thereafter. The cellular metabolic activity is likely to be at its optimum at 2 weeks healing since the bone morphological parameters are not stable and decreased thereafter.

This rabbit model encountered several limitations that jeopardise a full understanding of the effects of L-PRF treatment, such as the lack of fixation devices to stabilize the clot and potential bone damage due to high mobility of the animals especially after the 1st week. As mentioned above, the anatomy of the tibia does not favour retention of the L-PRF membrane. Because of the disruption of blood vessels in the defect, factors released from L-PRF only might have a paracrine effect. Hence, if the membrane is slightly displaced, the factors released by L-PRF are diluted over a volume that is larger than the created defect.

Furthermore, measuring bone morphometric parameters by μCT only indirectly assesses the strength, and hence the functionality of the bone. The results are sensitive to the selection of the regions of interest. Technologies using higher resolution are currently being developed and enable accurate measurements for bone microstructure in small volumes, and allow to adequately characterize the bone composition (discrimination between bone tissue and adipose tissue, cartilage and bone, vasculature, etc. via the use of contrast agents). Moreover, many μCT parameters show great variability between the individual rabbits, hampering statistical analysis. Further studies should therefore focus on accurate methods to describe, amongst others, bone mineral properties for cortical and medullary areas of non-critical sized bone defects. A further improvement could include a description of the permeability modification in these bone microenvironments, since this is likely an important parameter governing healing kinetics. Finally, the effects of L-PRF are expected to be more pronounced in a study that uses rabbits with systemic diseases that interfere in bone and wound healing, such as diabetes and osteoporosis.

Despite clear evidence of L-PRF-mediated bone healing in clinical studies and case reports, animal studies often fail to confirm this. Since the protocol for L-PRF production was mainly developed for human use, translation to other species is difficult. This leads to variation in protocols and experimental design, on top of the already present differences in biology and physiology.

In conclusion, L-PRF did not enhance bone tissue repair of non-critical size defects in rabbit tibia during our 4 week experiment. Its presence did promote microarchitectural changes related to connectivity network (Tb.Pf) during the healing process. Within the first 2 weeks, L-PRF had an adverse effect on the trabecular connectivity. This period is followed by accelerated improvement of the trabecular connectivity between 2 and 4 weeks. Similarly, mRNA expressions also suggest decreased healing dynamics at 14 days of healing when L-PRF is applied. The reasons for these observations are subject to further research.

## Additional Information

**How to cite this article**: Faot, F. *et al*. The effect of L-PRF membranes on bone healing in rabbit tibiae bone defects: micro-CT and biomarker results. *Sci. Rep.* **7**, 46452; doi: 10.1038/srep46452 (2017).

**Publisher's note:** Springer Nature remains neutral with regard to jurisdictional claims in published maps and institutional affiliations.

## Supplementary Material

Supplementary Table S1

## Figures and Tables

**Table 1:** Results of μCT analysis of bone regeneration at cortical bone level for L-PRF-treated and control groups, in function of healing time.

Healing time	Treatment	BV (mm^3^)	BV/TV (%)	BS (mm^2^)	BS/BV (mm^−1^)	BS/TV (mm^−1^)	i.S (mm^2^)	Tb.Pf (mm^−1^)	SMI	DA	FD
28 days	Control	5.20 (±0.85) A	58.33 (±9.61) A	77.85(±21.59) A	14.97 (±3.64) A	8.73 (±2.43) A	17.55 (±1.41) A	−11.44 (±6.82) A	−2.01 (±1.37) A	1.57 (±0.27) B	2.75 (±0.05) A
	L-PRF	5.38 (±1.60) A	60.47 (±17.96) A	70.30 (±12.13) A	13.94 (±4.25) A	7.90 (±1.36) A	17.10 (±1.54) A	−10.07 (±2.94) A	−2.38 (±2.01) A	1.83 (±0.77) B	2.76 (±0.07) A
14 days	Control	2.39 (±0.74) B	26.90 (±8.28) B	86.33 (±45.48) A	33.78 (±10.72) B	9.70 (±5.11) A	11.85 (±1.79) B	8.81 (±3.92) B a	2.92 (±0.74) B	1.30 (±0.16) A	2.54 (±0.05) C
	L-PRF	2.04 (±0.43) B	22.92 (±4.88) B	72.65 (±22.12) A	34.98 (±3.62) B	8.15 (±2.46) A	10.84 (±1.64) B	16.38 (±1.82) B b	3.98 (±0.44) B	1.19 (±0.08) A	2.47 (±0.01) C
7 days	Control	1.44 (±0.19) C	16.17 (±2.12) B	20.18 (±1.91) B	14.12 (±1.00) A	2.27 (±0.21) B	9.74 (±1.10) C	9.12 (±4.00) B	3.21 (±0.79) B	1.71 (±0.23) B	2.59 (±0.06) B
	L-PRF	1.43 (±0.17) C	16.13 (±1.90) B	19.97 (±2.48) B	13.93 (±0.89) A	2.24 (±0.28) B	9.92 (±0.99) C	7.75 (±4.37) B	2.93 (±1.29) B	1.71 (±0.20) B	2.61 (±0.04) B

Different capital letters within the same column indicates statistical significant differences between the healing time groups, irrespective to the treatment applied. Different lower case letters indicate the statistical significant differences found between the untreated (control) and treated (L-PRF) groups (*p* = 0.044).

**Table 2:** Results of μCT analysis of bone regeneration at medullar bone level for L-PRF-treated and control groups, in function of healing time.

Healing time	Treatment	TV (mm^3^)	BV (mm^3^)	BV/TV (%)	TS(mm^2^)	BS (mm^2^)	BS/BV (mm^−1^)	BS/TV (mm^−1^)	Tb.Th (mm)	Tb.Sp (mm)	Tb.N (mm^−1^)	i.S	Tb.Pf(mm^−1^)	SMI	DA	FD
28 days	Control	46.22 (±11.07) A	3.67 (±1.79) B	9.02 (±6.90) B	79.35 (±14.66) A	51.38 (±30.11) B	13.78 (±3.32) B	1.27 (±1.12) B	0.36 (±0.08) A	2.02 (±0.59) A	0.26 (±0.22) B	13.57 (±5.68) B	2.78 (±2.67) B	2.17 (±0.89) B	2.79 (±1.79) A	2.65 (±0.05) A
	L-PRF	47.48 (±10.91) A	2.56 (±1.18) B	5.52 (±2.50) B	81.18 (±14.60) A	41.73 (±15.01) B	17.93 (±6.47) B	0.91 (±0.38) B	0.30 (±0.09) A	2.00 (±0.41) A	0.19 (±0.11) B	12.46 (±3.44) B	5.84 (±10.65) B	2.16 (±1.77) B	2.33 (±0.81) A	2.56 (±0.16) A
14 days	Control	43.72 (±7.78) A	7.12 (±2.51) A	16.47 (±6.03) A	76.32 (±10.27) A	271.10 (±104.19) A	37.43 (±6.02) A	6.35 (±2.78) A	0.15 (±0.06) B	0.52 (±0.28) B	1.27 (±0.69) A	16.14 (±5.40) A	7.91 (±11.88) B	2.61 (±1.66) B	1.29 (±0.17) B	2.40 (±0.11) B
	L-PRF	47.12 (±11.16) A	7.78 (±2.10) A	16.98 (±4.86) A	80.70 (±14.87) A	318.83 (±103.91) A	40.82 (±8.08) A	7.18 (±3.20) A	0.16 (±0.07) B	0.60 (±0.42) B	1.40 (±0.95) A	19.37 (±4.45) A	3.35 (±6.33) B	2.00 (±0.63) B	1.22 (±0.07) B	2.45 (±0.08) B
7 days	Control	44.50 (±4.06) A	2.2 (±0.59) B	5.08 (±1.41) B	77.20 (±5.43) A	57.03 (±55.07) B	23.88 (±18.86) B	1.36 (±1.46) B	0.28 (±0.11) A	1.58 (±0.74) A	0.24 (±0.20) B	11.06 (±2.86) B	26.55 (±13.07) A	7.16 (±2.28) A	1.96 (±0.65) A	2.39 (±0.22) B
	LPRF	46.75 (±4.17) A	2.93±(0.97) B	6.39 (±2.61) B	80.20 (±5.55) A	60.52 (±37.42) B	19.37 (±6.06) B	1.34 (±0.93) B	0.30 (±0.08) A	1.46 (±0.45) A	0.25 (±0.21) B	13.54 (±2.85) B	26.72 (±7.21) A	8.15 (±2.03) A	1.82 (±0.33) A	2.45 (±0.09) B

Different capital letters within the same column indicate statistical significant differences between the healing time groups, irrespective to the treatment applied.
