# Supplementary material for: The effect of L-PRF membranes on bone healing in rabbit tibiae bone defects: micro-CT and biomarker results
Source: Sci Rep. 2017 Apr 12;7:46452. doi: 10.1038/srep46452 (PMC5388884; doi:10.1038/srep46452)
Supplement: Supplementary Table S1 [file srep46452-s1.doc]

**The effect of L-PRF membranes on bone healing in rabbit tibiae bone defects: micro-CT and biomarker results**

**Fernanda Faot, Sanne Deprez, Katleen Vandamme, Germana V. Camargos, Nelson Pinto, Jasper Wouters, Marc Quirynen, Joke Duyck**

**Supplementary Table**

Table S1. ANOVA results of the effect of the independent variables healing time, L-PRF vs no L-PRF application, and their interaction on the dependent variables (bone microstructural parameters) measured at the level of cortical bone and of medullar bone.

| **Cortical Bone** | | | | | **Medullar Bone** | | |
| --- | --- | --- | --- | --- | --- | --- | --- |
|  | | **Time** | **Treatment** | **Time [**x] **Treatment** | **Time** | **Treatment** | **Time [**x] **Treatment** |
| TV | ***x*** | | *x* | *x* | *p*=0.9107 | *p*=0.4359 | *p*=0.9565 |
| BV | ***p*<0.0001** | | *p*=0.5567 | *p*=0.6817 | ***p*<0.0001** | *p*=0.9174 | *p*=0.1795 |
| BV/TV | ***p*<0.0001** | | *p*=0.5673 | *p*=0.6740 | ***p*<0.0001** | *p*=0.8105 | *p*=0.8181 |
| TS | *x* | | *x* | *x* | *p*=0.9213 | *p*=0.4353 | *p*=0.9647 |
| BS | ***p*<0.0001** | | *p*=0.3091 | *p*=0.7336 | ***p*<0.0001** | *p*=0.6068 | *p*=0.7420 |
| i.S | ***p*<0.0001** | | *p*=0.3792 | *p*=0.6081 | ***p*=0.0075** | *p*=0.2899 | *p*=0.4253 |
| BS/BV | ***p*<0.0001** | | *p*=0.2783 | *p*=0.6845 | ***p*<0.0001** | *p*=0.0834 | *p*=0.7575 |
| BS/TV | ***p*<0.0001** | | *p*=0.901 | *p*=0.2550 | ***p*<0.0001** | *p*=0.8105 | *p*=0.8181 |
| SMI | ***p*<0.0001** | | *p*=0.7433 | *p*=0.2915 | ***p*<0.0001** | *p*=0.8227 | *p*=0.4940 |
| DA | ***p*<0.0001** | | *p*=0.5967 | *p*=0.4990 | ***p*=0.0001** | *p*=0.5455 | *p*=0.9822 |
| FD | ***p*<0.0001** | | *p*=0.5602 | *p*=0.0524 | ***p*=0.0013** | *p*=0.6347 | *p*=0.2975 |
| Tb.Pf | ***p*<0.0001** | | *p*=0.0853 | ***p*=0.0440** | ***p*<0.0001** | *p*=0.6980 | *p*=0.4836 |
| Tb.Sp | *x* | | *x* | *x* | ***p*<0.0001** | *p*=0.9122 | *p*=0.8937 |
| Tb.N | ***x*** | | *x* | *x* | ***p*<0.0001** | *p*=0.9703 | *p*=0.8618 |
| Tb.Pf | ***x*** | | *x* | *x* | ***p*<0.0001** | *p*=0.8871 | *p*=0.6069 |
